# Investigation of Parent Austenite Grains from Martensite Structure Using EBSD in a Wear Resistant Steel

**DOI:** 10.3390/ma10050453

**Published:** 2017-04-26

**Authors:** Jessica Gyhlesten Back, Göran Engberg

**Affiliations:** 1 Materials Science Division, Dalarna University, 791 88 Falun, Sweden; gen@du.se; 2 Research and Development, SSAB Europe, 781 84 Borlänge, Sweden

**Keywords:** dilatometry, hardness, EBSD, martensite, austenite, Kurdjumov-Sachs, 81.30.Kf

## Abstract

Crystallographic reconstruction of parent austenite grain boundaries from the martensitic microstructure in a wear resistant steel was carried out using electron backscattered diffraction (EBSD). The present study mainly aims to investigate the parent austenite grains from the martensitic structure in an as-rolled (reference) steel sample and samples obtained by quenching at different cooling rates with corresponding dilatometry. Subsequently, this study is to correlate the nearest cooling rate by the dilatometer which yields a similar orientation relationship and substructure as the reference sample. The Kurdjumov-Sachs orientation relationship was used to reconstruct the parent austenite grain boundaries from the martensite boundaries in both reference and dilatometric samples using EBSD crystallographic data. The parent austenite grain boundaries were successfully evaluated from the EBSD data and the corresponding grain sizes were measured. The parent austenite grain boundaries of the reference sample match the sample quenched at 100 °C/s (CR100). Also the martensite substructures and crystallographic textures are similar in these two samples. The results from hardness measurements show that the reference sample exhibits higher hardness than the CR100 sample due to the presence of carbides in the reference sample.

## 1. Introduction

Low alloy wear resistant martensitic steels are widely used in structural applications due to their excellent mechanical properties, especially high strength and wear resistant properties. Some of these steels suffer from flatness problems and residual stresses during hot rolling and quenching in the manufacturing process, which affects the mechanical properties of the end product. For steel producers it is important to have even quality of their steels to ensure reliability to customers. In order to reduce the residual stresses and avoid costly post processing to counter flatness problems, it is important to understand the history of deformation and the microstructure obtained from the manufacturing process [1]. The final microstructure of these steels after the final processing step in manufacturing is generally martensite and the mechanical properties depends on the microstructure [2]. In order to understand the martensitic microstructure, it is necessary to understand the prior austenitic morphology. The crystallographic orientation relationship (OR) is maintained when austenite transforms into martensite. This allows investigation of the austenitic phase by reconstruction of the crystallographic OR, which can be used to understand the mechanical properties [3].

In the present study, martensitic steel samples of Hardox 450 both as-received from the manufacturing process and dilatometry samples are characterized by way of electron backscatter diffraction (EBSD) and micro hardness measurements. The main aims of the investigation are to determine the austenite grain size and shape using EBSD data, with an algorithm to find the parent austenite grain boundaries, in order to understand the hardness results of a reference sample and two dilatometry samples. Thus, austenite grain size affects the size of martensite crystals and the size of parallel arrays of martensite crystals, and thereby affects the strength of hardened microstructures [4]. Subsequently, this study is to correlate the nearest cooling rate by a dilatometer which yields a similar orientation relationship and substructure as the reference sample. This knowledge will improve further experimental setup, modelling, and simulation of the hardening process.

Martensite is a hard phase in steels which forms when the steel is cooled rapidly from the austenitic region. Martensitic microstructures can also be generated by plastic deformation [5], for example, in metastable austenitic steels [3]. There are two types of martensite that can form, one is the cubic α’ which is a semi-coherent phase and the other is the hexagonal ε which is a coherent phase. The type of martensite depends on the transformation, the carbon content, and the stacking fault energy [6]. The formation of martensite in steels occurs via a diffusionless transformation which means that the carbon content within the crystal is trapped in octahedral sites of a body-centred cubic structure (bcc) [3]. The super-saturated carbon content affects the distortion of the new lattice and the crystal’s tetragonality [3]. Increasing carbon content will result in more tetragonality due to the high occupancy of carbon in the interstitial sites [7]. Carbon content also affects the martensitic microstructure that can develop in ferrous α’ martensitic bcc or body-centred tetragonal (bct) structures, i.e., lath-, lenticular plate-, thin plate-, and butterfly- martensites [8,9]. These martensitic transformations can be distinguished by morphology, kinetics, crystallography, or internal structure [6].

During martensitic transformation, the austenite crystals experience straining represented by a volume change as well as shear which changes the crystal structure. The already transformed martensitic crystals strain the remaining adjacent austenite crystals due to the increased volume of the harder martensite. Thus, the austenite experiences elastic and plastic deformation. The interface connecting the martensite with the austenite must have high mobility and therefore will move with high velocities during rapid quenching [3]. The phase boundary is either coherent or semi coherent, and an OR exists between the martensite and parent austenite phase. The OR is maintained by lattice distortions which results in the formation of dislocation cells or deformation twins, which reduce the system’s energy [10].

The OR between adjacent crystals in solid state is important in order to understand the material’s mechanical properties. Reproducible OR between the parent and product phases determines the morphology of martensite by reducing the interface energy and creating the best fit between the crystals. The OR usually consists of parallel or very nearly parallel corresponding closest packed planes from the two lattices, and it is usually the case that the corresponding close packed directions in these planes are also roughly parallel [3].

Many OR models have been developed for the austenite to martensite transformation, but the most commonly used are the Kurdjumov-Sachs (K-S), Nishiyama-Wasserman (N-W), Greninger-Troiano (G-T), and Bain OR models. Table 1 shows the planes and directions for the OR for these four models [11]. Gungunes et al. [12] found that austenite and thermally induced martensite usually have an OR of the Kurdjumov-Sachs type.

Lath martensite has a three-level hierarchy in its morphology, which is laths, blocks, and packets. For the K-S OR, 24 possible equivalent crystallographic variants can be possibly formed within a grain and six variants in a packet. However, Kitahara et al. [13] found that all variants do not appear in a grain. In sub-blocks, specific combinations of two variants appeared and this indicates a strict rule for variant selection. Variant selection is the result of shape deformation of a martensite lath/plate that may or may not comply with an external stress. The compliance of a variant and applied stress can be described by an interaction energy [14]. Positive interaction energy for a variant, which is in the plane subjected to an applied stress, means that the variant is favoured. Kundu et al. [15] showed that the position and space constraint in the microstructure might play a role in deciding which variants that will form regardless of their interaction energy. It is important to remember that the austenite almost always is constrained during quenching due to internal stresses because of temperature gradients in the material.

A martensite lath is a single martensite crystal. Lath boundaries usually have a misorientation of 2°–5°. Martensite laths (variants) with a similar crystal orientation pair up and build blocks [16]. The pairs are built by V1 and one of the variants from V2 to V6 (see Table 2), and the border between the pairs is the block boundary. Packets may consist of several blocks with slightly different orientations but the same habit plane [16]. Packet boundaries can have misorientation angles in both low-angle and high-angle intervals [17]. The effective grain structures which control deformation are the block and packet structures, because of the largely common crystallographic orientation of the parallel component laths within the blocks and packets [18]. When packet size decreases, the strength of low-carbon steels increases and affects the toughness of the martensitic steel [19].

Polycrystalline materials with a random distribution of grain orientations require that at least five independent strains can be achieved for two grains to be deformed without decohesion between the grains. Therefore, it requires at least five independent slip systems in each grain of a polycrystalline material. The five independent slip systems that result in the highest Schmid factors of the grain are used to calculate the Taylor factor m, as the inverted mean value [20].

In the custom build model, the Taylor factor was calculated for each pixel and a mean value for all were calculated. The slip systems used in the calculations were {10−1} <111> and {11−2} <111>. In bcc-metals there are usually many favourable glide systems which cause the Taylor factor to be close to the lowest value, which is two when all planes and directions are possible slip systems. This means that there are always several glide systems that have close to the maximum resolved shear stress. If the material has strong texture, the Taylor factor becomes dependent on the orientation, just as for a single crystal [20].

## 2. Materials and Methods

### 2.1. Method

The custom build Matlab™ (Mathworks, Natick, USA) model was used to calculate the local disorientation, which is the smallest misorientation angle for a common rotation axis, using the crystallographic orientation obtained by EBSD. The mean value of the disorientation to the left and below each pixel is the local disorientation. The evaluation method is based on the K-S OR between the parent austenite crystal and the daughter martensite. High angle grain boundaries (HAGB) have been evaluated for angles above 10° and low angle grain boundaries for disorientation angles above 2°.

Conditions for a martensite boundary is established on the basis of misorientation and the boundary must be considered as a parent austenite grain boundary when the condition is not fulfilled.

The martensite boundary here is defined as the boundary between the martensite variants of one parent austenite grain. The other boundaries, which do not fulfil the conditions, are boundaries between martensite laths of two different parent austenite grains and they are therefore considered as parent austenite grain boundaries.

The 24 possible variants of martensite orientation given by K-S OR for a single austenite grain result in 276 possible combinations of variants with corresponding misorientation angles and rotation axes between adjacent martensite crystals.

All HAGBs were then compared to the possible misorientation angles and the corresponding rotation axes, and they were considered as martensite boundaries if they agreed within ±10 degrees. The rest of the boundaries were considered to be parent austenite grain boundaries according to the EBSD analysis.

The results for the misorientation angle and misorientation axis in this work, shown in Table 2, are compared with the results of Kitahara et al. [13]. These specific misorientations are between martensite variants of one parent austenite grain. Therefore the parent austenite grain boundaries can be determined by searching for the boundaries between martensites with misorientations which are different from those specific values.

### 2.2. Material

The samples were taken from the abrasion resistant steel, Hardox 450 (a trade name from SSAB, Sweden) for the present study. These steels generally have a fully martensitic structure after hot rolling and quenching with a unique combination of high hardness and high strength with excellent impact toughness.

The slab, of 220 mm thickness, is heated to about 1250 °C before entering the hot rolling mill, where it is rolled to a strip with 6 mm thickness. When the strip reaches the finishing mill its temperature is about 1000 °C and the exit temperature of the strip is 890 °C. The reduction in this finishing mill begins at 30% and reduces to 12% at the exit. The material is fully recrystallized at the exit. The lowest recrystallization temperature is approximated to be 884 °C from the equation developed by Barbosa et al. [21]. However, this equation involves the influence of micro alloying elements, which however are not present in Hardox 450, and which can impede recrystallization [22]. The strip is then water quenched in the cooling section.

The chemical composition of these samples is shown in Table 3. A reference sample was taken from the final coiled product which had undergone hot rolling and quenching. Samples from Hardox 450, manufactured in the same way as the reference sample, have been subjected to various cooling rates by use of dilatometry.

### 2.3. Dilatometry and Micro Hardness

The dilatometric experiments were performed in a quenching dilatometer DIL 805A from TA instruments (New Castle, DE, USA). The samples were 4 mm diameter cylinders with 10 mm length that had been cut from the centre of the strip’s width of a 6 mm thick Hardox 450 strip in the rolling direction. The surfaces were parallel and flat. The samples were first heated to 890 °C at 10 °C/s and then austenitised for 2 min with subsequent cooling to room temperature. The cooling rates used were 60 °C/s and 100 °C/s. The samples will hereafter be called CR60, CR100, and reference. The CR60 and CR100 samples were chosen for EBSD analysis due to the formation of fully martensitic structures.

To elucidate the effect of carbide formation during cooling and the state of the reference sample, dilatometry tests were performed in two cycles using the same heating and cooling as for CR100. Six samples were subjected to cyclic testing. Three of them had no hold time at room temperature (RT) and the others had 2 min hold time at RT.

Micro Vickers hardness measurements were performed on the reference sample and the dilatometry samples using a Matsuzawa MXT 50 (Akita Prefecture, Akita, Japan) automatic digital micro hardness tester with 500 g load.

### 2.4. EBSD-Analysis

The EBSD measurements were performed using a field emission gun Zeiss Ultra 55 scanning electron microscope (Carl Zeiss Microscopy, Jena, Germany). Data acquisition and post-processing of crystalline data were performed using HKL Channel 5 software (version 5.12.61.0, Oxford Instrument NanoAnalysis, Oxford, UK). The step size used for each sample was 0.2 μm. The area for EBSD analyses was chosen to be 100 μm wide and about 80 μm high, and the surfaces were normal to the rolling direction and in the vicinity of the centre of the specimen.

## 3. Results and Discussion

### 3.1. EBSD Measurements

**Figure 1:**
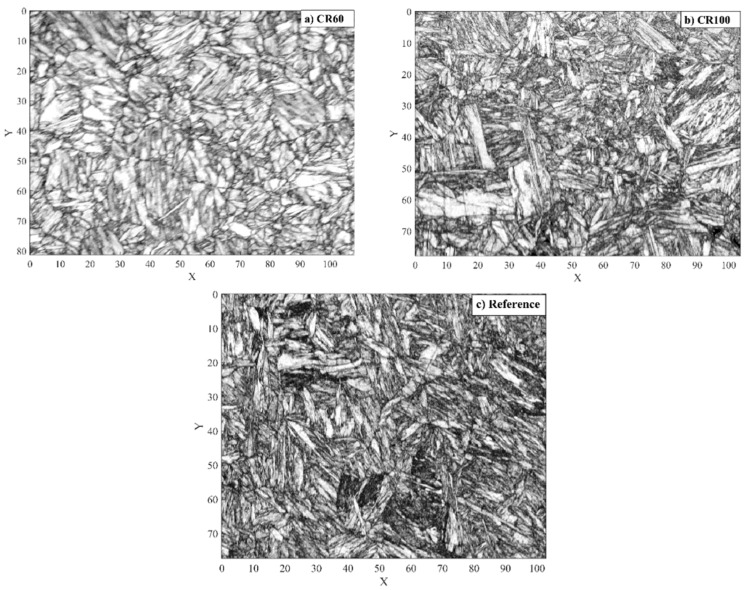
The band contrast images from the EBSD analysis for (**a**) CR60, (**b**) CR100 and (**c**) the reference sample in rolling direction with the axis showing size in μm.

Figure 1a–c shows the band contrast images for the reference, CR60, and CR100 samples obtained from the EBSD analyses which reveal fully martensitic microstructures for all of the samples. The CR100 and reference samples are relatively similar and their band contrasts show a distorted microstructure, which indicates strong deformation caused by the phase transformation i.e., martensitic lath structure and substructure. CR100 and the reference sample have finer laths and more substructure as compared to the CR60 sample. This can be attributed to differences in plastic deformation of the austenite due to temperature gradients combined with the successive growth of martensite from the cooled surface. A lower cooling rate gives lower temperature gradients and consequently the austenite in the centre of the specimen is less constrained due to the martensite formation as compared to the surface.

Table 4 shows the list of calculated results of the Taylor factor in three perpendicular directions *x*, *y*, and *z* for the applied stress. The Taylor factor for these samples shows that there is no strong texture [23] that would give different properties in different directions. There is no significant difference in the Taylor factors for the three samples.

### 3.2. Evaluation of EBSD Data

**Figure 2:**
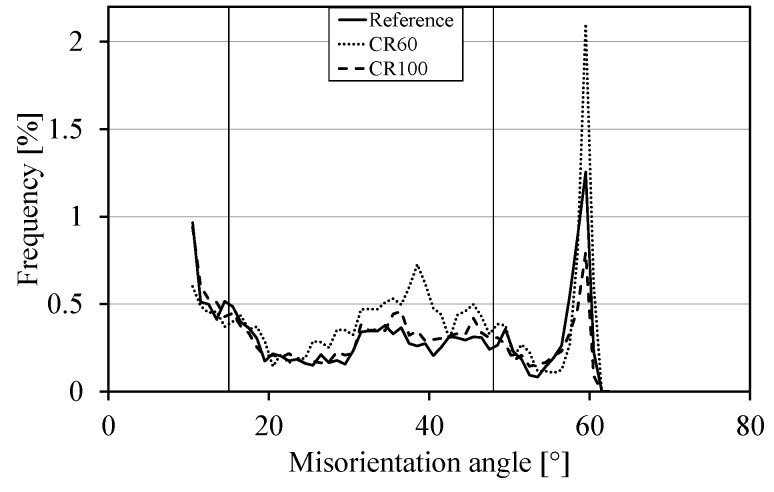
The frequency of parent austenite boundaries for all samples versus the misorientation for martensite boundaries.

The data from the EBSD measurements are evaluated using the K-S OR. All boundaries with a misorientation angle above 10° were checked for being a possible martensite boundary allowing 10° deviation from the martensite boundary misorientation angle and axis. All other boundaries are supposed to be parent austenite boundaries. Figure 2 shows the frequency of parent austenite grain boundaries with the misorientation angle (of martensite boundaries) of all the samples. The plot for CR100 and the reference samples follow a similar pattern.

**Figure 3:**
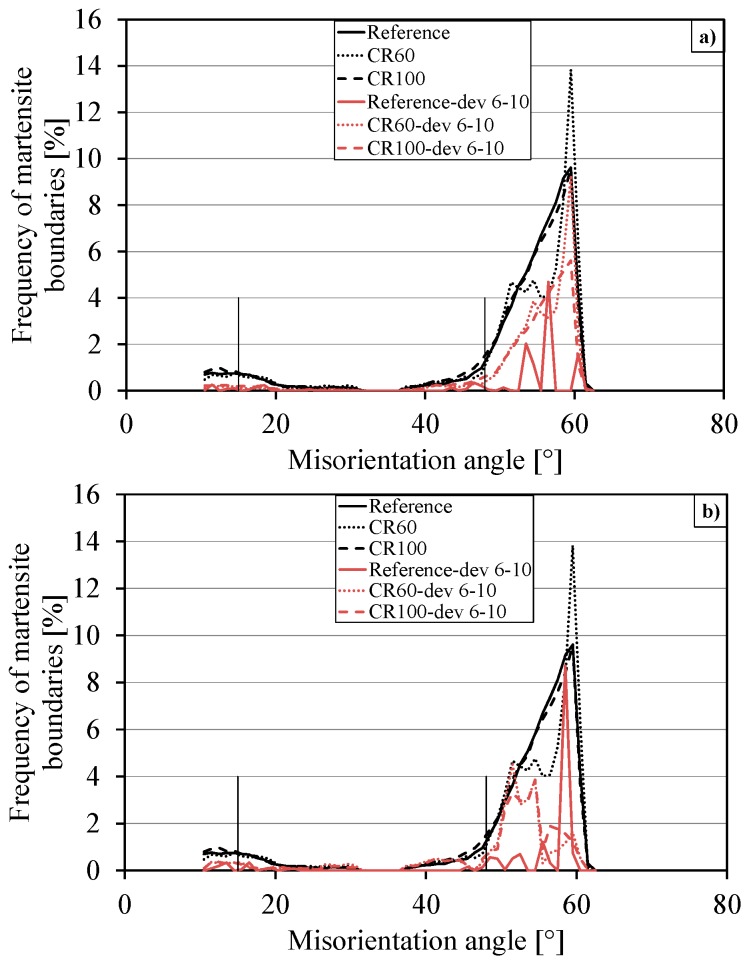
The frequency distribution of martensite boundaries and their deviation from (**a**) the axis and (**b**) the angle for the misorientation angle between 10.5° and 63°.

The model handles misorientations between adjacent grains and describes them by rotation axis and by rotation angle. Figure 3 shows the axis and angle deviations for respective samples.

In Figure 3a, it can be seen that CR100 and the reference sample have a similar frequency of martensite boundaries according to the axis deviation, and the same conclusion can be drawn from Figure 3b where the angle deviation reveals a similar distribution for the CR100 and reference samples. However, CR60 deviates from CR100 and the reference sample. As can be seen from Figure 3, the frequency of observed martensite boundaries for respective samples have the lowest values between 15° and 48°. Some amount of information was lost when the boundaries with misorientations lower than 10° were not counted.

**Figure 4:**
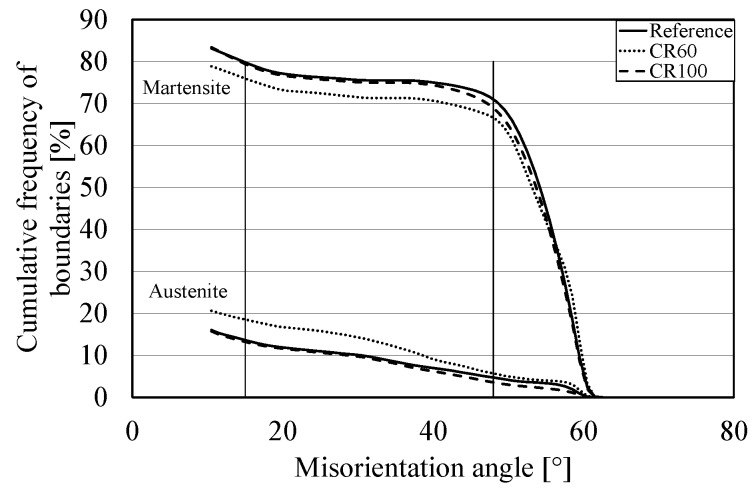
The curves show the cumulative frequency of martensite boundaries for misorientation angles between 10.5° and 63°. The higher curves indicate the martensite boundaries and the lower curves show the parent austenite boundaries.

Figure 4 also shows that the reference and CR100 samples are similar for the case of the cumulative frequency of boundaries, both martensitic and parent austenitic boundaries.

It can be observed from Figure 4 that the cumulative frequency of martensite boundaries is mainly observed above 48° and below 15°. The reference sample contains slightly more martensite boundaries than CR100, and CR60 has the lowest amount. When it comes to austenite boundaries, the reference sample and CR100 have the same amount. The above results reveal that the cooling rate 100 °C/s results in a similar microstructure to the reference sample.

**Figure 5:**
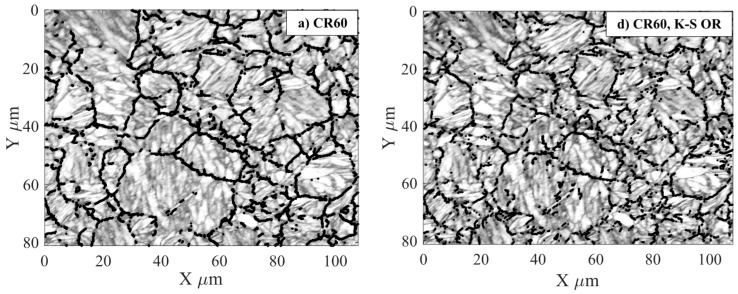

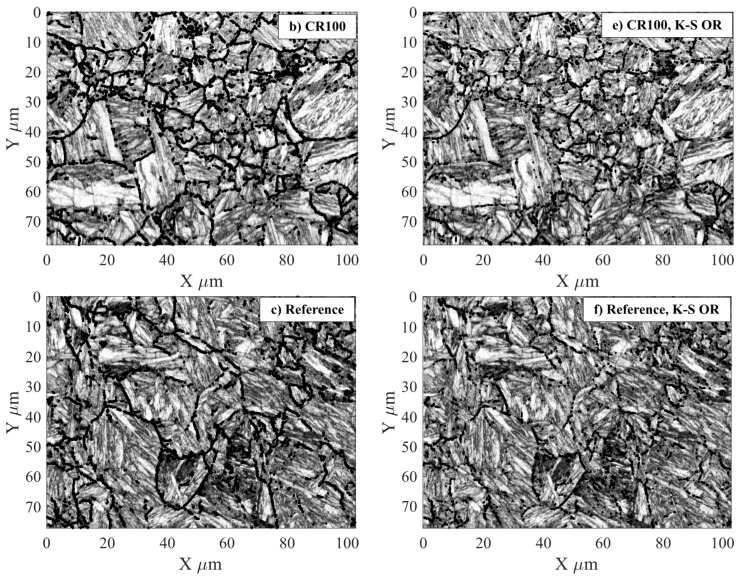
The martensite boundaries that are marked with black colour in the band contrast image represents parent austenite boundaries. (**a**–**c**) show misorientations between 15°–48°; (**d**–**f**) show the Kurdjumov-Sachs orientation relationship (±10°).

Figure 5a–c shows assumed parent austenite grains with the misorientations between 15° and 48° which are marked with black colour for each sample in the band contrast images. The boundaries between different variants of orientation-relations according to K-S have been determined by comparing HAGB with the variants that K-S predicts in respect to misorientation and the rotation of the axis.

**Figure 6:**
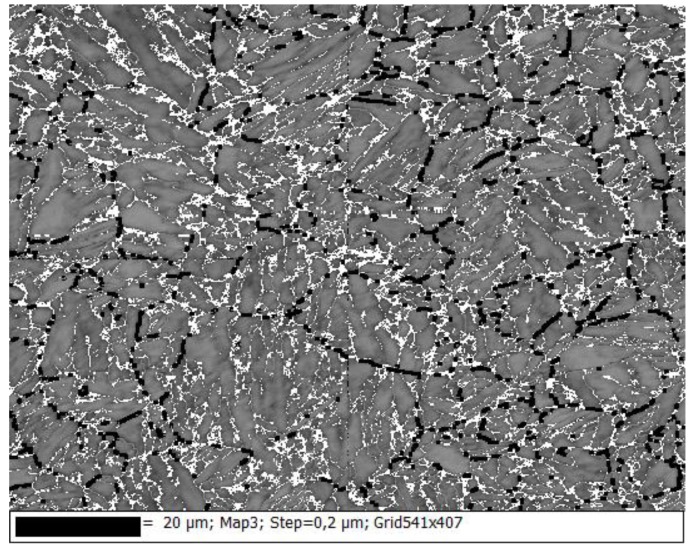
Band contrast of the CR60 sample with the parent austenite boundaries in black and the zero solutions in white.

Figure 5d–f shows the parent austenite grains, also drawn in black, according to the Kurdjumov-Sachs OR ±10° for all samples. Results show that boundaries within 15° to 48° angles are parent austenite grain boundaries due to their grain size and shape. Other misorientation angle ranges (10°–55°) have been tried but misorientation angles from 15° to 48° yield the best fit for the grain boundaries and the results observed in Figure 4 also confirm the same. For all samples, the black lines do not intercept everywhere. This occurs around the areas that are not indexed, and locations where no Kikuchi pattern can be found (see Figure 6).

Comparing the images in Figure 5a–c, which show band contrasts and parent austenite boundaries when they are drawn using misorientation angles of 15°–48°, with Figure 5d–f, showing band contrast and OR K-S for the respective samples, reveals that choosing this misorientation angle range agrees well with the boundaries evaluated by the K-S OR. No flattening of the parent austenite grains can be seen in any direction for all the samples, thus full recrystallization was reached before quenching.

**Figure 7:**
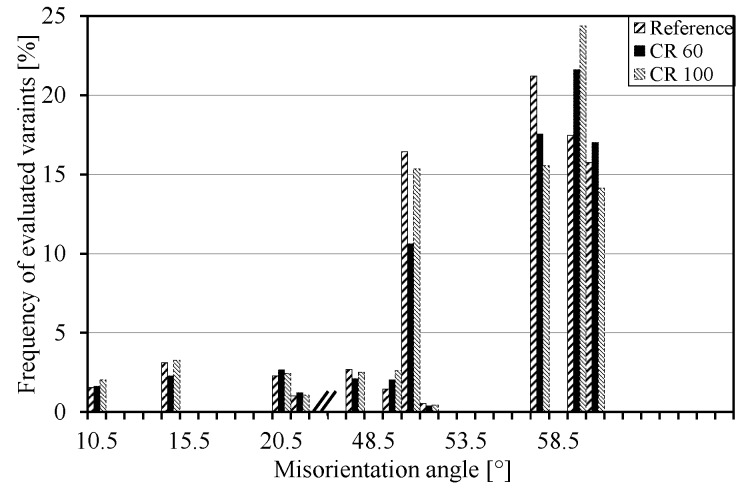
The frequency of the evaluated variants in each sample.

Figure 7 shows the frequency of evaluated variants plotted according to their misorientation angle. The variants in this plot cannot be separated by their numbers since some variants have the same misorientation angle. The reference and the CR100 sample show similar trends for misorientations below 55° while the CR60 sample deviates from this trend. Above 55°, no clear trend is visible. Table 2 shows the list of crystal orientations of 24 martensite variants transformed from a (001)[100] oriented austenite grain maintaining the Kurdjumov-Sachs OR {(111)//(011) and [−101]//[−1−11]} and crystallographic relationships between V1 and other variants.

**Figure 8:**
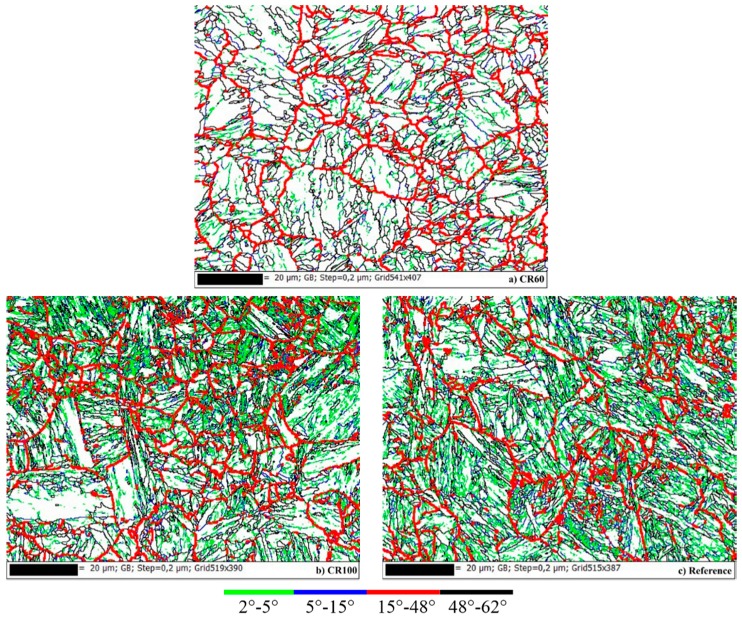
Boundaries with misorientations from 2°–62° are drawn in (**a**) CR60, (**b**) CR 100 and (**c**) the reference sample.

In Figure 8a-c, the boundaries have been drawn with different colours in order to illustrate the substructure for each sample. Noise reduction has been applied to the data by the nearest neighbour method with three neighbours and by removing wild spikes. Lath boundaries can be observed between 2°–5° which are drawn with green colour. As can be seen in Figure 8b,c, a large fraction of green areas in the CR100 sample and in the reference sample reveals more lath boundaries. Green boundaries are also the boundaries of subgrains inside the martensite lath. Between 15°–48°, parent austenite grain boundaries can be observed for all investigated samples which are presented with red colour. Black boundaries are the block and packet boundaries. The blue colour shows possible lath, packet, and austenite boundaries.

The CR60 sample shows a significantly less distorted misorientation distribution in the area where the majority of martensite boundaries are observed. The EBSD images also clearly show a coarser substructure in this sample compared to the other two. The substructure has been studied by viewing the boundaries where the misorientation is ≥2°.

The austenite grain size varies in each sample, ranging from 5 to 20 microns in diameter. Black boundaries are block and packet boundaries, which are observed between 48°–62°. The mean grain size has been measured to be 12 µm for CR60, 11 µm for CR100, and 17 µm for the reference sample. However, the number of grains are not enough to ensure good statistical significance.

Table 5 lists the measured sizes of grains having a misorientation larger than 2°. This was done with HKL Channel 5 software, which calculated the average size, standard deviation, and minimum and maximum values of the grains.

These values clearly show that the substructure of the reference sample and CR100 have similar sizes of their subgrains. CR60 has about 0.5 μm larger subgrains. However, these values also show that the average grain size for all samples fall within the measurement uncertainty.

### 3.3. Texture Analysis

**Figure 9:**
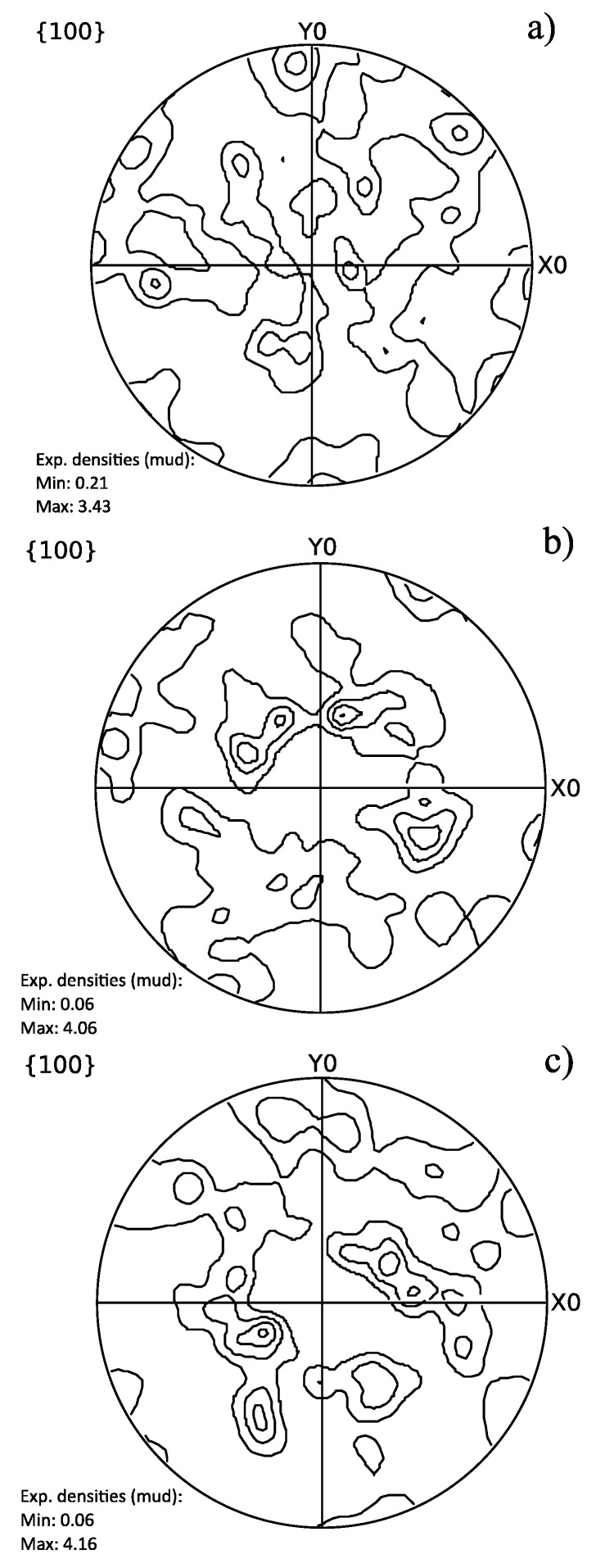
{100} pole figures of (**a**) CR60; (**b**) CR100 and (**c**) Reference samples with the rolling direction parallel to Z0.

Figure 9 shows the pole figures for the different samples which reveal weak textures. Also the Taylor factor reveals that the mechanical properties are similar in all directions. Comparing the samples, with both band contrast images (Figure 1) and pole figures (Figure 9), clearly shows similar orientations between CR100 and the reference samples. The pole figures for CR100 and the reference sample can be rotated around the rolling direction (Z0) to match one another since cylindrical samples are used where the normal and transverse direction of the sheet is not known. However, CR60’s pole figure does not have a matching pattern regardless of how the pole figure is rotated.

### 3.4. Dilatometry and Hardness Measurements

**Figure 10:**
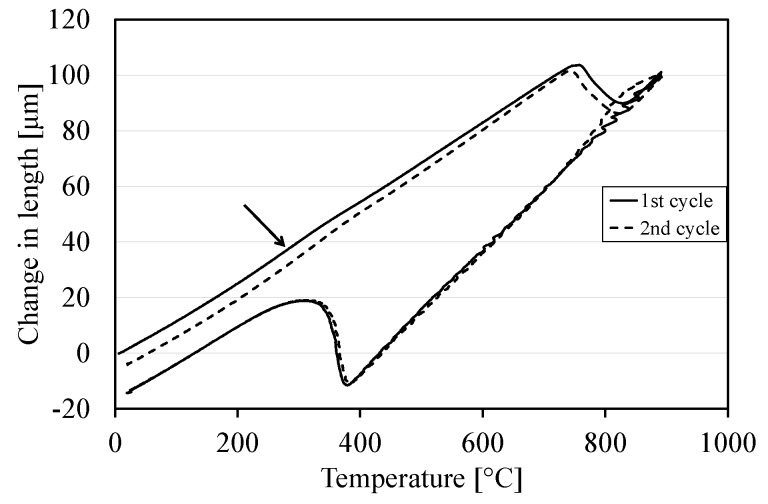
Dilatometry curves for a sample heat-treated in two cycles.

Figure 10 shows the dilatation curves for one CR100 sample heat treated in the dilatometer in two cycles. The heating and cooling was performed exactly the same in both cycles, with no hold time at room temperature. The same trend was observed at heating for all three samples tested. The cooling curves were adjusted to match each other so that the variation during heating could be visible. It can be observed that there is a slight reduction in slope in the first cycle as compared to the second cycle at around 300 °C, as shown by an arrow in Figure 10. This can be correlated to the presence of carbides in the as-received condition, which is thus the same state as the reference sample in this trial.

**Figure 11:**
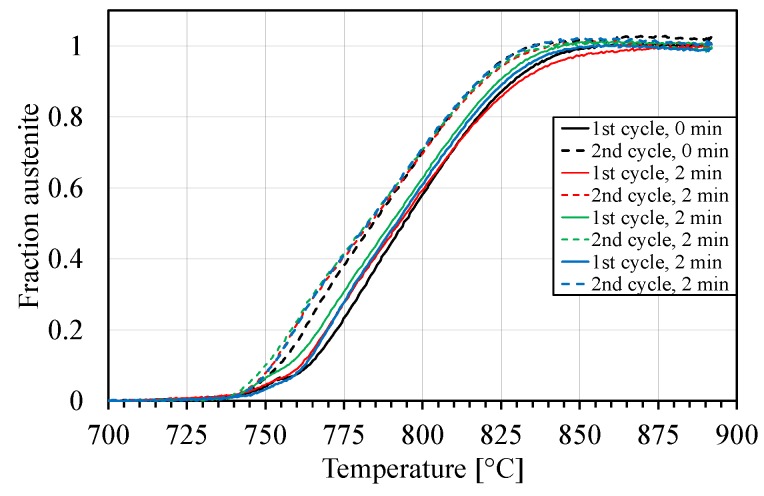
The fraction of austenite formation during the two cycle heat treatment.

Figure 11 shows the amount of austenite formed during heating in the first and second cycle for some of the tested samples. The lever rule with a linear relationship was used to plot these curves for the dilatation between 600 to 720 °C during heating and 700–500 °C during cooling.

It can be observed in Figure 11 that the rate of austenite transformation at early stages is lower in the first cycle than in the second. After about 5%–10% transformation, the conversion rates will be approximately the same. A very reasonable explanation for this behaviour is that carbides (probably very small) are formed after coiling of the hot rolled as-received steel. The growth of carbides starts when the temperature reaches about 300 °C, which means that the carbon in solid solution decreases, resulting in a decreased dilatation. The carbides impede the transformation to austenite until they are dissolved. Christian [24] has reviewed the strength of martensite and effectively related it to the structural changes produced by the lattice and lattice-invariant deformations characterized by the crystallographic theory of martensite transformation. He also found that carbon diffusion cannot be suppressed in low-alloy steels and iron-carbon alloys, and in order to generate useable high-strength microstructures, it is even promoted by low-temperature tempering. Winchell and Cohen [25] found that the yield stress of freshly quenched martensite may be increased by low temperature ageing, during which the precipitation of carbides takes place. Moreover, autotempering can occur during the quench of carbon steels with a martensite start temperature, Ms, above RT which means that precipitates are formed during the quench; in some low carbon steels, the precipitates have been identified as cementite [18]. It follows that some strength of martensite may be caused by the clustering of carbon or by the precipitation of carbides during quenching. This should be regarded as a conventional strengthening mechanism because (a) the transformation enables a high supersaturation to be achieved; (b) the precipitation is effected at a low temperature, so that a fine dispersion is formed, and (c) the precipitation occurs in a phase which possesses an extraordinarily complex substructure. Transition-iron-carbides (ε) dominate the microstructure of the tested 4340 steel in the study made by Thompson [26]. There is also some evidence that cementite, however small, is present in the microstructure. Hexagonal Fe_3_C and Fe_2_C iron-carbides are called epsilon-carbides, and orthorhombic iron-carbides are referred as eta-carbides.

In the second cycle there is no time for carbides to form before the temperature starts to increase. Thus, there are no available nuclei for carbide formation at 300 °C and carbide formation does not occur. Later when the transformation to austenite begins, there are no carbides which can impede the transformation. Figure 11 shows the transformation curves of one sample without hold time at RT and three samples with two minutes hold time at RT.

**Figure 12:**
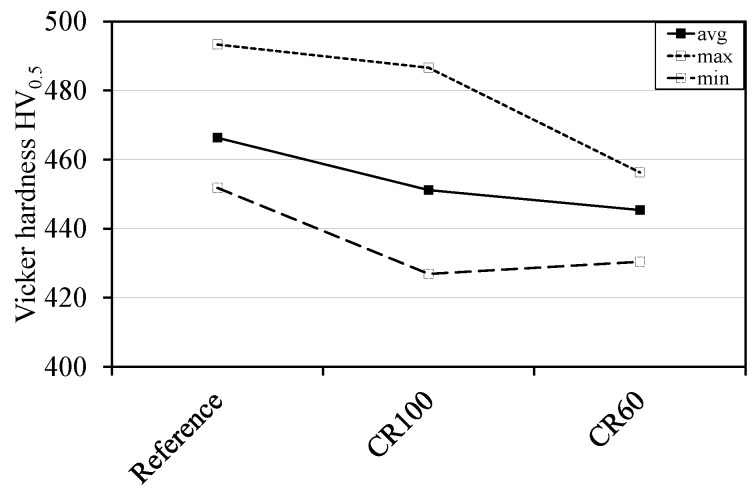
Average hardness in the centre of the respective samples.

These results indicate that the presence of carbides affects the formation of austenite and also the mechanical properties of the steel, for example, the hardness of the reference and dilatometry samples as shown in Figure 12. This figure shows differences in hardness between the reference and the dilatometry samples. The hardness values decrease from the reference condition to dilatometry samples due to presence of more carbides in the reference sample. Martensite hardness depends on several factors, including the structure’s fineness (grain size), dislocation density (mean free path for dislocation glide), the amount of carbon in solid solution, subsequent segregation of carbon to dislocations, and carbide precipitation [27].

The hardness difference between the reference sample and CR100 can be attributed to the carbides, whereas the difference between the reference sample and CR60 is the effect of the carbides and the coarser substructure, i.e., a larger mean free path for dislocation slip.

## 4. Conclusions

The low alloyed wear resistant steel shows fully martensitic microstructures in both reference and dilatometry samples.

One main difference between the reference sample and the dilatometry samples are the amount of existing carbides, which affects the hardness. The reference sample and CR100 have a similar distorted microstructure, fine substructure, and crystallographic texture, while CR60 has a coarser microstructure which also contributes to a lower hardness. The dilatometry sample CR60 shows a less distorted misorientation distribution in the area where the majority of martensite boundaries are observed. The EBSD images also show coarser substructure in this sample as compared to the other two.

The reconstruction of parent austenite grain boundaries with K-S OR between martensite and austenite shows that the dilatometry sample cooled with 100 °C/s is almost identical to the reference sample when it comes to the different types of HAGB (≥10° of misorientation).

The data from the EBSD measurements are evaluated using the K-S OR. The frequency of evaluated parent austenite boundaries for respective samples is most frequent between 15° and 48°. The comparison between different misorientation angles shows that 15°–48° yields the most logical visual image of the parent austenite grains for Hardox 450. The reference sample and CR100 reveal similar misorientation patterns, which also means that the two have similar parent austenite grain structure.

The reconstructed parent austenite grain boundaries in the reference sample showed no significant flattening due to rolling, thus implying a fully recrystallized structure before quenching. Similar austenite grain sizes (HAGB) were observed in all samples. The substructure in CR100 and the reference sample were of equal size with respect to low angle grain boundaries.

The main aims of this study were to determine the austenite grain size and shape, and also to understand the hardness results of the reference sample and the dilatometry samples. This has been accomplished. Also, this study has correlated the nearest cooling rate by the dilatometer which yields similar OR and the substructure as the reference sample. This work contributes to knowledge about the correlation between the austenitic structure, martensitic structure, and the conditions after quenching.

## Figures and Tables

**Table 1:** Commonly used orientation relationship (OR) models used to describe the relation between austenite and martensite in steels, from [11].

OR	Plane	Direction
Kurdjumov-Sachs (K-S)	${\left\{111\right\}}_{\gamma }//{\left\{110\right\}}_{\alpha }$	${\langle 110\rangle }_{\gamma }//{\langle 111\rangle }_{\alpha }$
Nishiyama-Wasserman (N-W)	${\left\{111\right\}}_{\gamma }//{\left\{110\right\}}_{\alpha }$	${\langle 011\rangle }_{\gamma }//{\langle 001\rangle }_{\alpha }$ or ${\langle 112\rangle }_{\gamma }//{\langle 110\rangle }_{\alpha }$
Greninger-Troiano (G-T)	${\left\{111\right\}}_{\gamma }\sim 1{}^{\circ}//{\left\{110\right\}}_{\alpha }$	${\langle 12,\;17,\;5\rangle }_{\gamma }//{\langle 17,\;17,\;7\rangle }_{\alpha }$
Bain	${\left\{010\right\}}_{\gamma }//{\left\{010\right\}}_{\alpha }$	${\langle 001\rangle }_{\gamma }//{\langle 101\rangle }_{\alpha }$

**Table 2:** Crystal orientations of 24 variants from the Kurdjumov-Sachs orientation relationship and crystallographic angle/axis relationships between V1 and other variants.

Variant	Austenite OR K-S	Martensite OR K-S	Misorientation Angle from V1	Misorientation Axis to V1		
1	(111)[−101]	(011)[−1−11]	–	–	–	–
2	(111)[−101]	(011)[−111]	60.00	0.577	−0.577	0.577
3	(111)[01−1]	(011)[−1−11]	60.00	0	−0.707	−0.707
4	(111)[01−1]	(011)[−11−1]	10.53	0	−0.707	0.707
5	(111)[1−10]	(011)[−1−11]	60.00	0	0.707	0.707
6	(111)[1−10]	(011)[−11−1]	49.47	0	0.707	0.707
7	(1−11)[10−1]	(011)[−1−11]	49.47	−0.577	−0.577	0.577
8	(1−11)[10−1]	(011)[−11−1]	10.53	0.577	−0.577	0.577
9	(1−11)[−1−10]	(011)[−1−11]	50.51	−0.186	0.767	0.615
10	(1−11)[−1−10]	(011)[−11−1]	50.51	−0.490	−0.463	0.739
11	(1−11)[011]	(011)[−1−11]	14.88	0.354	−0.933	−0.065
12	(1−11)[011]	(011)[−11−1]	57.21	0.357	−0.714	0.603
13	(−111)[0−11]	(011)[−1−11]	14.88	0.933	0.354	0.065
14	(−111)[0−11]	(011)[−11−1]	50.51	−0.739	0.463	0.490
15	(−111)[−10−1]	(011)[−1−11]	57.21	−0.246	−0.628	−0.738
16	(−111)[−10−1]	(011)[−11−1]	20.61	0.659	0.659	0.363
17	(−111)[110]	(011)[−1−11]	51.73	−0.659	0.363	−0.659
18	(−111)[110]	(011)[−11−1]	47.11	−0.302	−0.626	−0.719
19	(11−1)[−110]	(011)[−1−11]	50.51	−0.615	0.186	−0.767
20	(11−1)[−110]	(011)[−11−1]	57.21	−0.357	−0.603	−0.714
21	(11−1)[0−1−1]	(011)[−1−11]	20.61	0.955	0	−0.296
22	(11−1)[0−1−1]	(011)[−11−1]	47.11	−0.719	0.302	−0.626
23	(11−1)[101]	(011)[−1−11]	57.21	−0.738	−0.246	0.628
24	(11−1)[101]	(011)[−11−1]	21.06	0.912	0.410	0

**Table 3:** Chemical composition of the test material Hardox 450.

Element	C	Si	Mn	P	S	Cr	Ni	Mo	B
wt %	0.19	0.70	1.60	0.025	0.010	0.25	0.25	0.25	0.004

**Table 4:** The calculated mean values of the Taylor factor are listed for each sample.

Sample	Taylor Factor		
	*x*	*y*	*z*
CR60	2.42	2.46	2.40
CR100	2.51	2.46	2.50
Reference	2.49	2.48	2.43

**Table 5:** The statistics of all boundaries with misorientations above 2°.

Specimen	CR60	CR100	Reference
Average (μm)	2.0262	1.4285	1.4457
Standard deviation (μm)	1.6693	1.0989	1.0921
Minimum value (μm)	0.71365	0.71365	0.71365
Maximum value (μm)	15.056	13.001	10.369
Size of the data set (μm)	1393	2219	2300

## Abbreviations

EBSD	Electron Backscatter Diffraction
OR	Orientation Relationship
K-S	Kurdjumov-Sachs
CR	Cooling rate
